# 4-1BB Signaling in Conventional T Cells Drives IL-2 Production That Overcomes CD4^+^CD25^+^FoxP3^+^ T Regulatory Cell Suppression

**DOI:** 10.1371/journal.pone.0153088

**Published:** 2016-04-06

**Authors:** Hampartsoum B. Barsoumian, Esma S. Yolcu, Haval Shirwan

**Affiliations:** Institute for Cellular Therapeutics and Department of Microbiology and Immunology, University of Louisville, Louisville, Kentucky, 40202, United States of America; University of South Carolina School of Medicine, UNITED STATES

## Abstract

Costimulation with the recombinant SA-4-1BBL agonist of 4-1BB receptor on conventional CD4^+^ T cells (Tconvs) overcomes the suppression mediated by naturally occurring CD4^+^CD25^+^FoxP3^+^ T regulatory cells (Tregs). The mechanistic basis of this observation has remained largely unknown. Herein we show that Tconvs, but not Tregs, are the direct target of SA-4-1BBL-mediated evasion of Treg suppression. IL-2 produced by Tconvs in response to 4-1BB signaling is both necessary and sufficient for overcoming Treg suppression. Supernatant from Tconvs stimulated with SA-4-1BBL contains high levels of IL-2 and overcomes Treg suppression in ex vivo Tconv:Treg cocultures. Removal of IL-2 from such supernatant restores Treg suppression and repletion of Tconv:Treg cocultures with exogenous recombinant IL-2 overcomes suppression. This study establishes 4-1BB signaling as a key circuit that regulates physical and functional equilibrium between Tregs and Tconvs with important implications for immunotherapy for indications where a fine balance between Tregs and Teffs plays a decisive role.

## Introduction

CD4^+^CD25^+^FoxP3^+^ T regulatory cells (Tregs) play a critical role in tolerance to self-antigens and are involved in acquired tolerance in settings of tumors, infections, and transplantation [1,2]. Modulation of Treg frequency and/or function, therefore, has important therapeutic implications for various diseases. Treg cells express various costimulatory receptors belonging to both CD28 [3] and tumor necrosis factor (TNFR) [4–6] families of receptors, and they require costimulation for their generation, proliferation, and long-term maintenance [3]. The CD28 costimulatory molecule plays a critical role in Treg generation and function [3]. However, the role and importance of costimulatory members of the TNFR family in modulating Treg responses remain to be fully investigated.

The 4-1BB receptor of the TNFR family is one of the costimulatory molecules constitutively expressed on Tregs [4,5,7]. Studies focusing on the role of 4-1BB pathway in immune regulation relied on the use of agonistic Abs to the receptor. Given the significant in vivo functional differences reported between agonistic Abs and the natural ligands, particularly with respect to the ability of the Abs to bind and modulate FcγRs [8], we generated a novel form of 4-1BB ligand, referred to as SA-4-1BBL, by cloning the extracellular functional domain of the ligand to the C-terminus of a modified streptavidin core [6]. This molecule exists as oligomers and has robust T cell costimulatory activity in soluble form [9,10]. As component of tumor associated subunit vaccines, SA-4-1BBL demonstrated robust therapeutic activity in various tumor models [9–15]. Therapeutic efficacy was associated with the ability of SA-4-1BBL to generate robust Th1 responses, overcome CD8^+^ T cell anergy, and increase intratumoral CD8^+^T/Tregs ratio [10–15].

Previous work in our lab has demonstrated that SA-4-1BBL was effective in expanding Tregs stimulated through TCR and IL-2 without altering their suppressive function [6]. We also showed that costimulation with SA-4-1BBL overcomes Treg suppression of Tconvs [6]. Subsequent studies confirmed that evasion of suppression was not due to direct engagement of SA-4-1BBL with its receptor on Tregs, but on Tconvs [15]. The major focus of this study is to elucidate the mechanistic basis of this observation. The data presented herein demonstrate that SA-4-1BBL-stimulated production of IL-2 in Tconvs is necessary and sufficient for overcoming Treg suppression.

## Materials and Methods

### Mice and reagents

Transgenic C57BL/6.Foxp3^GFP^ and wild type C57BL/6 mice were purchased from The Jackson Laboratory. Human CD2 transgenic C57BL/6.Foxp3^hCD2^ [16] and 4-1BB deficient C57BL/6.4-1BB^-/-^ mice [17] were bred and cared for in University of Louisville’s specific-pathogen-free vivarium in accordance with NIH guidelines. All animal procedures were approved by the University of Louisville’s IACUC. Antibodies used include anti-CD3 agonistic Ab (clone 145-2C11, BD Pharmingen, Cat# 553058), anti-TGF-β (1,2,3) neutralizing Ab (clone 1D11, homemade), anti-IFN-γ blocking Ab (clone XMG 1.2, homemade), and anti-IL-2 neutralizing Ab (clone S4B6, BD Pharmingen, Cat# 554375). Fluorescently-labeled Abs to various cell surface markers were obtained commercially: α-CD4-APC (BD Pharmingen, 553051); α-CD25-PE (BD Pharmingen, 553866); α-CD25-PE-Cy7 (eBioscience, 25-0251-82); α-hCD2-PE (BioLegend, 300208). Where indicated Tconvs or Tregs were stained with 2.5μM carboxyfluorescein succinimidyl ester (CFSE) (Invitrogen, C1157). CTLL-2 cell line was obtained from ATCC (TIB-214). Human IL-2, SA-4-1BBL, and SA proteins were produced in our lab according to standard protocols as previously reported [10,11].

### Flow cytometry cell sorting

Both Tconvs and Tregs were sorted from splenocytes of naive C57BL/6.Foxp3^GFP^ mice using BD FACSAria by gating on CD4^+^ CD25^-^ GFP^-^ cells to collect Tconvs and on CD4^+^ CD25^+^ GFP^+^ population to collect Tregs. Tconvs from WT C57BL/6 or Tregs from C57BL/6.Foxp3^hCD2^ mice were sorted by gating on CD4^+^ CD25^-^ to collect Tconvs and CD4^+^ CD25^+^ to collect Tregs. Sorted populations purity was ≥ 99%. In select experiments, Tregs were expanded in vitro using an agonistic anti-CD3 Ab, SA-4-1BBL, and IL-2 as described [6].

### Suppression assay

Freshly sorted Tconvs (2.5 x 10^4^) were cocultured with freshly sorted or expanded Tregs (2.5 x 10^4^) and irradiated (2000 cGy) naive C57BL/6 splenocytes (1 x 10^5^, 2000 cGy) as APCs in 96-titer U-bottom plates. All suppression assays were conducted using sorted Tconvs and Tregs from C57BL/6.Foxp3^GFP^ mice, except where Tconvs and Tregs were labeled with CFSE. In the latter case, Tconvs were sorted from wild type C57BL/6 and Tregs from hCD2 transgenic C57BL/6.Foxp3^hCD2^ mice. Cultures were supplemented with anti-CD3 Ab (0.5 μg/ml), SA-4-1BBL (1 μg/ml), and IL-2 (3 IU/ml) where indicated and maintained for 48 h at 37°C in a 5% CO_2_ incubator. Cultures were pulsed with 1 μCi/well of [^3^H]-thymidine (PerkinElmer, Cat# NET027X001MC) for an additional 16 h, harvested, and counts per minute (CPM) were recorded and used as a measure of cell proliferation.

### Removal of SA-4-1BBL and IL-2 from Tconv culture supernatant

Tconvs were stimulated with anti-CD3 Ab and SA-4-1BBL protein for 48 h at 37°C in a 5% CO_2_ incubator, culture supernatants were harvested and then incubated with CuSO_4_-charged sepharose beads to remove the 6xHis tagged SA-4-1BBL. For removal of IL-2, culture supernatant was incubated with an anti-IL-2 Ab (clone S4B6; 20 μg/ml) at 37°C for 15–30 minutes and Ab/IL-2 complexes were removed by incubating with Immobilized Protein G high affinity beads (Thermo Scientific, Cat# 20398).

### Cytometric bead array

The level of various cytokines (IL-2, IL-4, IL-6, IFN-γ, TNF-α, IL-17a) in cell culture supernatant was quantified using a cytometric bead array assay following the manufacturer's instructions (BD Biosciences, Cat# 560485).

### RT-PCR for IL-2 expression

Tconvs and Tregs were sorted from splenocytes of naive C57BL/6.Foxp3^GFP^ mice using BD FACSAria by gating on CD4^+^ CD25^-^ GFP^-^ cells to collect Tconvs and on CD4^+^ CD25^+^ GFP^+^ population to collect Tregs. Cocultures were stimulated with SA-4-1BBL (1 μg/ml) and plate-bound anti-CD3 Ab (5 μg/ml). Cultures were harvested for total RNA isolation 24 and 48 hours post-stimulation. RNA was converted into cDNA and used for real-time PCR amplification using primers for housekeeping gene glyceraldehyde 3-phosphate dehydrogenase (GAPDH) and IL-2. Primer sequences were as follows: IL-2 forward 5’-TGA GCA GGA TGG AGA ATT ACA GG-3’, reverse 5’-GTC CAA GTT CAT CTT CTA GGC AC-3’; GAPDH forward 5’-CCA TCA CCA TCT TCC AGG AGC GAG-3’, reverse 5’-CAC AGT CTT CTG GGT GGC AGT GAT-3’. Delta-Delta CT values were calculated and normalized to GAPDH.

### Statistical analysis

Data represent mean ± SEM. The statistical significance was estimated with two-tailed Student’s *t* test using GraphPad software. A *p* value ≤ 0.05 was considered statistically significant.

## Results

### APCs do not contribute to SA-4-1BBL-mediated evasion of Treg suppression

**Fig 1:**
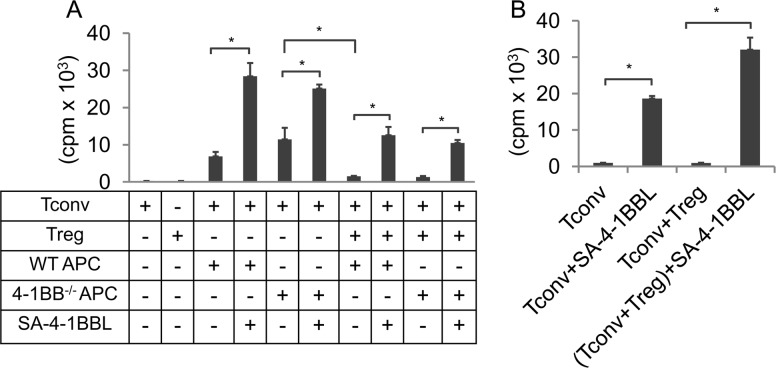
Costimulation by SA-4-1BBL overcomes suppression of Tconvs by Tregs independent of APCs. **(A)** Flow-sorted Tconvs were cocultured with freshly sorted or expanded Tregs at 1:1 ratio in the presence of irradiated splenocytes from wild type or 4-1BB^-/-^ mice as APCs and anti-CD3 Ab (0.5 μg/ml). SA-4-1BBL (1 μg/ml) was added to cultures where indicated. Cultures were incubated for 48 h, pulsed with [^3^H]-thymidine for an additional 16 h, and proliferation was measured and graphed as cpm. **(B)** Suppression assay was conducted with sorted Tconvs and Tregs without APCs using plate bound anti-CD3 Ab (5 μg/ml) and soluble SA-4-1BBL (1 μg/ml) where indicated. Each data point is indicative of mean ± SEM of triplicate wells and representative of 2 separate experiments. Student’s t-test (two-tailed) was performed for statistical analysis with *p ≤ 0.05 being significant.

We recently demonstrated that costimulation with SA-4-1BBL protein allows Tconvs to overcome Treg suppression in ex vivo coculture studies that included irradiated antigen presenting cells (APCs) [15]. APCs, particularly DCs, express 4-1BB and signaling via this receptor results in upregulation of immunostimulatory molecules and cytokines [10,15,18,19]. Therefore, we first tested if 4-1BB signaling in APCs contributes to Tconv evasion of Treg suppression. Coculture experiments were performed in the presence of irradiated splenocytes from wild type and 4-1BB knockout (KO) mice as APCs. Addition of SA-4-1BBL to the culture overcame inhibition of sorted Tconv proliferation by Tregs, irrespective of the presence or absence of 4-1BB expression by APCs (Fig 1A).

To further confirm that APCs do not play a role in the Tconv evasion of Treg suppression, the coculture studies were performed in the absence of APCs. Stimulation of Tconvs with plate bound anti-CD3 Ab alone under these culture conditions did not generate a significant proliferative response (Fig 1B). However, costimulation with SA-4-1BBL resulted in robust Tconv proliferation that was refractory to suppression by Tregs. Taken together these data demonstrate that costimulation via 4-1BB receptor expressed on APCs is not involved in SA-4-1BBL-mediated Tconv resistance to suppression by Treg cells.

### TGF-β or IFN-γ are not involved in SA-4-1BBL-mediated evasion of Treg suppression

**Fig 2:**
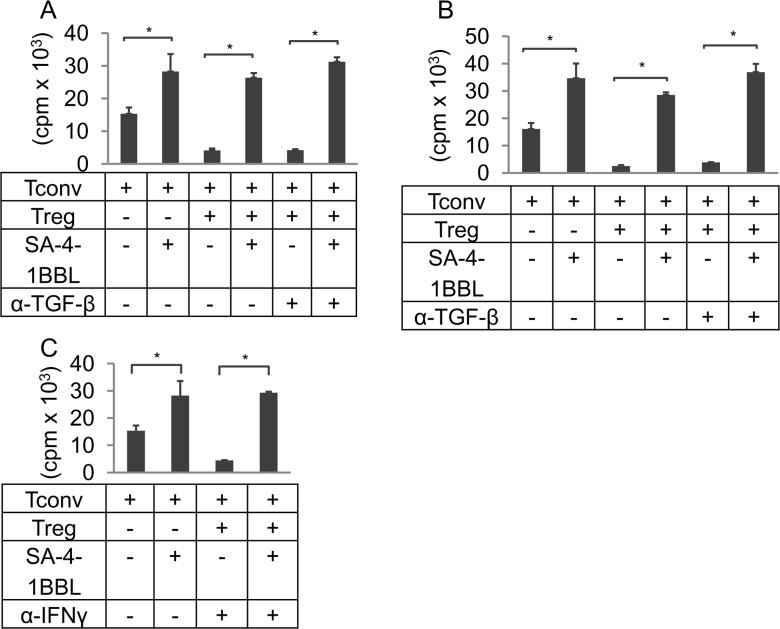
TGF-β and IFN-γ are not involved in Tconv evasion of Treg suppression. Sorted Tconvs and Tregs were cocultured at 1:1 ratio in the presence of irradiated APCs and anti-CD3 Ab (0.5 μg/ml). Cultures were also supplied with SA-4-1BBL (1 μg/ml) and blocking Abs to TGF- (either 8 or 50 μg/ml) or IFN- (25 μg/ml) as shown. **(A)** Cultures supplemented with the blocking anti-TGFβ Ab at the time of incubation. **(B)** Cultures supplemented with Tregs pre-incubated with anti-TGF-β Ab (50 μg/ml). **(C)** Cultures supplemented with a blocking anti-IFN-γ Ab. Each data point in the bar graphs is indicative of mean ± SEM of triplicate wells and representative of 2 independent experiments. Student’s t-test (two-tailed) was performed for statistical analysis with *p ≤ 0.05 being significant.

TGF-β plays an important role in the generation as well as suppressor function of Tregs [20–22]. It has been shown that 4-1BB signaling in Tconvs modulates TGF-β1-mediated suppression [23]. We, therefore, asked if stimulation with SA-4-1BBL overcomes the Treg-mediated suppression by modulating the function of TGF-β. A neutralizing TGF-β Ab used at two different doses (8 and 50 μg/ml) did not prevent Treg suppression in the Treg:Tconv coculture experiments nor did it modulate the SA-4-1BBL-mediated evasion of Treg suppression (Fig 2A).

It has been demonstrated that T cells produce the active form of TGF-β in response to TCR triggering, which is adsorbed to the cell surface immediate post-secretion [24]. Membrane-bound TGF-β is a mediator of Treg suppression [25]. Therefore, we conducted studies to rule out the possibility that SA-4-1BBL may overcome Treg suppression by modulating the function of membrane bound/adsorbed TGF-β. Sorted Tregs were pre-incubated with a high dose of Ab (50 μg/ml) to block the function of membrane-bound/absorbed TGF-β. These cells were able to suppress Tconvs in cocultures studies and costimulation with SA-4-1BBL was still able to overcome suppression (Fig 2B).

An interplay between IFN-γ and TGF-β pathways was shown to regulate the suppression of CD4^+^ Tconvs [26]. Importantly, IFN-γ has been involved in the generation and function of Tregs [27,28]. We, therefore, tested the contribution of this cytokine in the observed evasion of Treg suppression. A blocking Ab against IFN-γ used at a high dose (25 μg/ml) did not improve the SA-4-1BBL-mediated evasion of suppression nor did it affect the suppression profile (Fig 2C). Collectively, these data demonstrate that TGF-β and IFN-γ, two cytokines involved in the modulation of Tconv and Treg functions, are not involved in SA-4-1BBL-mediated evasion of Treg suppression.

### IL-2 is the predominant cytokine produced in the supernatant of Tconv:Treg cocultures costimulated with SA-4-1BBL

**Fig 3:**
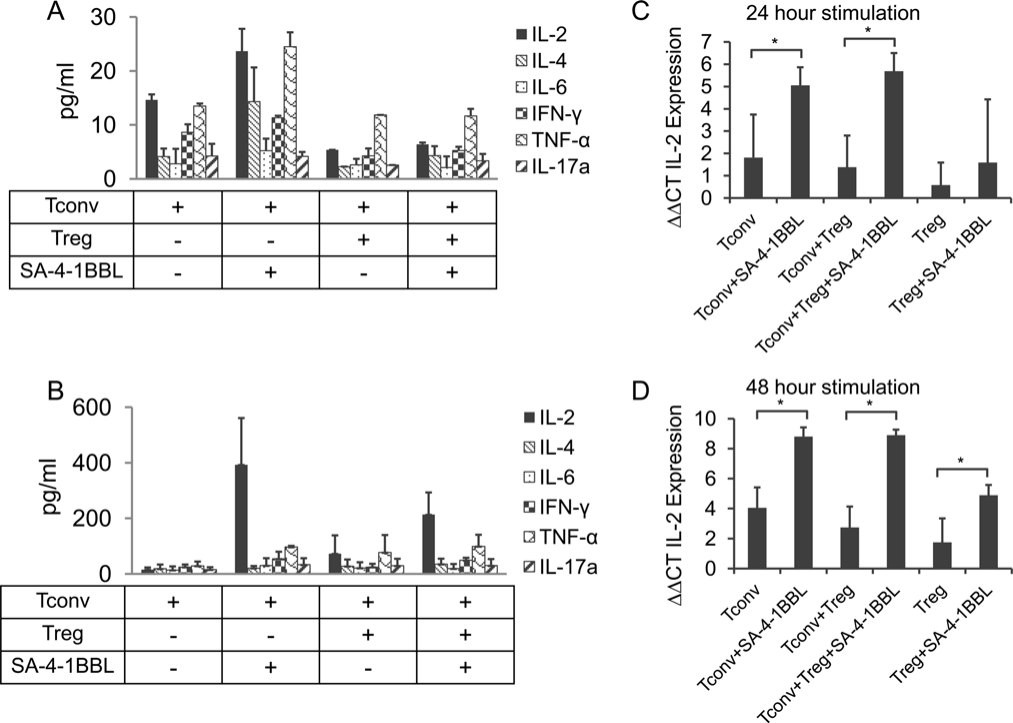
IL-2 is the predominant cytokine upregulated in Tconv and Tregs cocultures costimulated with SA-4-1BBL. Sorted Tconvs and Tregs were cocultured at 1:1 ratio and 48 h later supernatants were collected and subjected to cytometric bead array analysis. **(A)** Tconv:Treg cocultures containing irradiated APCs, SA-4-1BBL (1 μg/ml) as shown, and soluble anti-CD3 Ab (0.5 μg/ml). **(B)** Tconv:Treg cocultures without APCs, but including plate bound anti-CD3 Ab (5 μg/ml) and SA-4-1BBL (1 μg/ml) as indicated. Data shown in (**A** and **B**) is combination of two independent experiments with mean ± SEM reported. **(C, D)** RT-PCR ΔΔCT values for relative IL-2 mRNA expression with respect to GAPDH gene in indicated cultures stimulated with SA-4-1BBL (1 μg/ml) and plate-bound anti-CD3 Ab (5 μg/ml). IL-2 expression was assessed at 24 **(C)** and 48 **(D)** hours post-stimulation. Each data point in (**C** and **D**) represents the mean of triplicate wells ± SD, with *p ≤ 0.05 being significant.

We next tested if the analysis of T cell cultures stimulated with SA-4-1BBL might identify soluble factor(s) potentially responsible for the observed evasion of Treg suppression. As shown in Fig 3A, costimulation of Tconvs with SA-4-1BBL resulted in upregulated secretion of almost all cytokines tested. Notably, IL-2 and TNF-α were two cytokines whose production appreciably increased in the 48-hr culture supernatants. However, the levels of these two cytokines were similar in supernatants harvested from Tconv and Treg cocultures with or without SA-4-1BBL costimulation. This cytokine pattern was not time dependent, as 24-hr culture supernatants showed a similar pattern (data not shown).

The presence of APCs and particularly their irradiation status may impact overall cytokine production and presence in coculture supernatants. Therefore, these studies were performed with sorted Tconvs and Tregs in the absence of APCs. IL-2 stood out as the only cytokine with increased levels in supernatants harvested from Tconv (~400-folds) and Tconv:Treg (~200-folds) cocultures costimulated with SA-4-1BBL, but not in culture supernatants without SA-4-1BBL costimulation (Fig 3B). The mechanism(s) regulating the low levels of IL-2 produced in cultures with irradiated APCs are not known. However, the non-specific binding of IL-2 to the heterogeneous population of irradiated APCs, consumption by such cells, or other mechanisms, such as B7 on APCs cross-linking CTLA-4 molecules on T cells, thereby down-regulating IL-2 production by Tconvs [29,30], may provide an explanation.

We also noticed a significant drop in the levels of IL-2 protein in Tconv:Treg cocultures stimulated with SA-4-1BBL (Fig 3A and 3B) relative to stimulated cultures of Tconvs alone. To test if this is due to IL-2 produced by Tconvs being consumed by Tregs that do not produce significant levels of this cytokine, we performed quantitative RT-PCR to assess the level of IL-2 mRNA under various culture conditions. As shown in Fig 3C and 3D, IL-2 transcript levels were comparable between Tconv and Tonv:Treg cocultures stimulated with SA-41BBL examined at 24 and 48 hr time points. Taken together, these data suggest that the reduced levels of IL-2 protein observed in supernatant of Tconv:Treg coculture stimulated with SA-4-1BBL is not due to the diminished IL-2 transcript levels, but perhaps because of the consumption of this cytokine by Tregs.

### IL-2 is required for SA-4-1BBL-mediated Tconv evasion of Treg suppression

**Fig 4:**
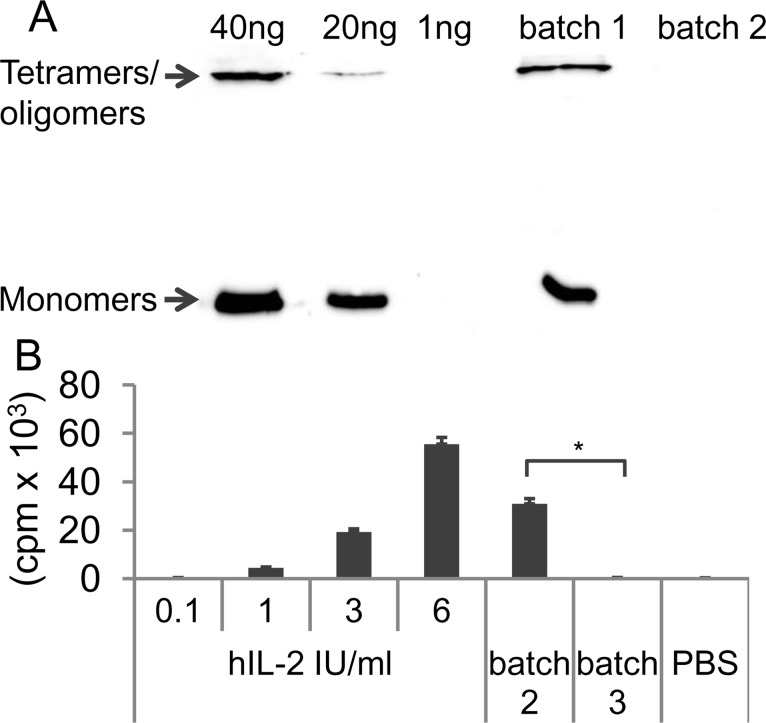
Removal of SA-4-1BBL and IL-2 from Tconv culture supernatants. **(A)** Supernatant (sup) of Tconvs stimulated with an agonistic anti-CD3 Ab (5 μg/ml) and SA-4-1BBL (1 μg/ml) were incubated with CuSO_4_-charged sepharose beads to remove SA-4-1BBL carrying a 6xHis tag. Regular sup (batch 1) and SA-4-1BBL-depleted sup (batch 2) were analyzed using anti-SA antibodies for detection in Western blots. The indicated amounts of SA-4-1BBL protein were used as detection controls. **(B)** Batch 2 supernatant described in (A) was incubated with an anti-IL-2 Ab (20 μg/ml) followed by the removal of Ab/IL-2 complexes using immobilized protein G beads to generate batch 3. Batch 2 as well as batch 3 supernatants (50 μl/each) were tested on the IL-2-dependent CTTL-2 cell line with the indicated commercial recombinant IL-2 doses (IU/ml) as positive and PBS as negative controls. Cultures were incubated for 28 h with [^3^H]-thymidine added for the last 8 h. Proliferation was measured and graphed as cpm. Each data point is indicative of mean ± SEM of triplicate wells. Student’s t-test (two-tailed) was performed for statistical analysis with *p ≤ 0.05 being significant. The data is representative of two independent experiments.

The high levels of IL-2 production in Tconv and Treg cocultures costimulated with SA-4-1BBL led us to directly test its role in the evasion of Treg suppression. Towards this end, SA-4-1BBL and IL-2 were depleted from culture supernatant from Tconvs stimulated with anti-CD3 and SA-4-1BBL. SA-4-1BBL was depleted using CuSO_4_ charged sepharose taking the advantage of a 6xHis tag engineered into the protein for purification purposes [6]. IL-2 was removed using an excess amount of anti-IL-2 Ab and protein G beads. The complete removal of SA-4-1BBL (Fig 4A) and IL-2 (Fig 4B) from culture supernatants was confirmed by western blots and CTLL-2 proliferation assay, respectively.

**Fig 5:**
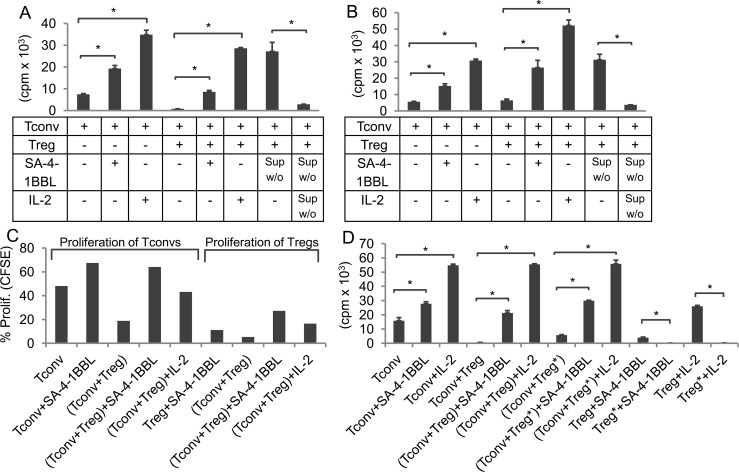
SA-4-1BBL costimulation-mediated IL-2 production by Tconvs is both necessary and sufficient in overcoming Treg suppression. Sorted Tconvs and Tregs were cocultured at 1:1 ratio in the presence **(A)** or absence **(B)** of irradiated APCs and anti-CD3 Ab. Cultures were also supplemented with SA-4-1BBL (1 μg/ml), IL-2 (3 IU/ml), SA-4-1BBL-depleted supernatants (100 μl/well), or SA-4-1BBL- and IL-2-depleted supernatants (100 μl/well) where indicated. **(C)** Tconvs sorted from WT C57BL/6 or Tregs sorted from C57BL/6.Foxp3^hCD2^ mice were labeled with 2.5μM CFSE and used in coculture suppression assays with irradiated APCs. Cultures were incubated for 64 h and cells were analyzed using flow cytometry for proliferation. **(D)** Tregs were irradiated at 2000 cGy (Tregs*) and used in coculture experiments mimicking panel **A**. Experiments were repeated twice with similar patterns. Student’s t-test (two-tailed) was performed for statistical analysis with *p ≤ 0.05 being significant.

Addition of SA-4-1BBL-free culture supernatant resulted in effective evasion of Treg suppression in Tconv:Treg cocultures with (Fig 5A) or without APCs (Fig 5B). In marked contrast, culture supernatant lacking both SA-4-1BBL and IL-2 was ineffective in overcoming Treg suppression (Fig 5A and 5B). The critical role of IL-2 in overcoming Treg suppression was further confirmed by supplementing Tconv and Treg cocultures with exogenous recombinant IL-2 (Fig 5A and 5B).

### IL-2-mediated Tconv evasion of Treg suppression is uncoupled from Treg proliferation

Both Tconvs and Tregs were shown to expand in response to inflammatory insults [31], suggesting that under such conditions Tconvs evade the suppression by Tregs. Although the excessive production of IL-2 by Tconvs could play an important role for the evasion of Treg suppression [32], it is also likely that actively proliferating Tregs temporarily lose their suppressive function. Indeed, Treg suppression is associated with a non-proliferative, anergic state [33]. Therefore, we have stained either Tconvs sorted from C57BL/6 or Tregs sorted from C57BL/6.Foxp3^hCD2^ splenocytes with CFSE prior to addition to suppression assays (Fig 5C). The CFSE labeled Tconvs proliferated by 67.5% with SA-4-1BBL and by 64.1% in the presence of Tregs and SA-4-1BBL. The CFSE labeled Tregs on the other hand proliferated by 11.1% with SA-4-1BBL and by 27.3% in the presence of Tconvs and SA-4-1BBL (Fig 5C). This may suggest that the proliferative state of Tregs could, in part, account for their lack of suppression in our experimental setting.

To eliminate the contribution of Treg proliferation to the observed evasion of suppression achieved by SA-4-1BBL, coculture experiments were performed using irradiated Tregs. As shown in Fig 5D, irradiated Tregs were functional, as they inhibited the proliferation of Tconvs. This inhibition was totally negated by the addition of SA-4-1BBL or recombinant IL-2 to cultures. These effects were similar to those obtained using non-irradiated Tregs, demonstrating that SA-4-1BBL-mediated Tconv evasion of Treg suppression requires IL-2 and is independent of the proliferative state of Tregs.

## Discussion

Previous studies have demonstrated pleiotropic functions of SA-4-1BBL in immune regulation [9]. In particular, SA-4-1BBL blocked the conversion of Tconvs into Tregs cells [34] and overcame Treg suppression of Tconvs [15]. SA-4-1BBL-stimulated IFN-γ expression in Tconvs was responsible for blocking their conversion into Tregs [34]. However, the mechanistic basis of SA-4-1BBL-mediated Tconv resistance to Treg suppression remains to be elucidated. The present study focused on this aspect and reports that SA-4-1BBL-mediated production of IL-2 by Tconv is required and sufficient for resistance to Treg suppression.

Tregs and a subpopulation of DCs constitutively express the 4-1BB receptor, while most other immune cells, such as Tconvs, NK cells, and macrophages, upregulate expression following activation by various means [9]. Therefore, the inhibitory effect of SA-4-1BBL on Treg function reported in this study may be direct, i.e. by targeting Tregs, or indirect by targeting Tconvs or APCs used in coculture experiments. Previous studies using cells from 4-1BB KO mice demonstrated that Tregs are not the direct target of SA-4-1BBL for the observed inhibition of suppressor function [15]. Stimulation of DCs by agonists of 4-1BB receptor may result in secretion of various cytokines, including IL-6, IL-12, and TNF-α [35,36] that may overcome suppression and contribute to the Tconv proliferation. However, the findings in the present study confirm our previous observation that 4-1BB costimulation overcomes Treg suppression of Tconvs [6,15] and further demonstrate that this effect is independent of 4-1BB signaling in APCs. APCs from 4-1BB KO mice did not affect SA-4-1BBL-mediated Tconv resistance to Treg suppression. Importantly, SA-4-1BBL conferred Tconv resistance to Treg suppression in the complete absence of APCs.

In an attempt to understand the mechanism of evasion, we initially hypothesized that if Tregs primarily suppress through TGF-β, SA-4-1BBL might be downregulating TGF-β receptor II on Tconvs, hence desensitizing them to suppression [37]. A neutralizing Ab to TGF-β under the specified coculture conditions did not overcome Treg suppression nor did it impact the SA-4-1BBL-mediated Tconv resistance to Treg suppression. These findings are in accordance with published studies demonstrating that thymically derived (t)Tregs suppress through cell-to-cell contact without a significant contribution by TGF-β [33,38]. Signaling via 4-1BB receptor on Tconvs results in IFN-γ production that has the ability to block TGF-β and tumor-mediated conversion of Tconvs into peripheral (p)Tregs [34]. Conversely, another study showed that IFN-γ contributes to the conversion of Tconvs into pTregs and improves their suppressive function by upregulating FoxP3 expression [37]. These opposing observations may reflect different experimental settings as one study used TGF-β and antigen stimulation-driven conversion, whereas the other study employed an antigen- and TGF-β-independent conversion setting. In the present study, a blocking Ab to IFN-γ neither prevented Treg suppression in the absence of SA-4-1BBL, nor altered SA-4-1BBL-mediated evasion of suppression. These observations are consistent with previous findings that IFN-γ has no direct effect on tTreg development and function [39].

IL-2 expressed by Tconvs is required and sufficient for the ability of these cells to evade Treg suppression under the tested coculture conditions. First, IL-2 was the major cytokine upregulated in cocultures stimulated with SA-4-BBL in the absence of APCs. Second, supernatant from SA-4-1BBL-stimulated cultures was effective in overcoming Treg suppression, and removal of IL-2 nullified this effect. Third, exogenous recombinant IL-2 alone was effective in overcoming Treg suppression. IL-2 is a growth factor and master regulator of both Tconv and Treg responses. Tregs express minimal levels of IL-2, and as such are dependent on Tconvs as the major source of IL-2. IL-2 upregulates the expression of FoxP3 and CD25, the α chain of the high affinity IL-2R, in Tregs [40,41]. Competition for IL-2 has been shown to be an important mechanism that dictates the functional balance between Tconvs and Tregs. Tregs express high affinity IL-2R and effectively compete with Tconvs for the available IL-2 [42]. Therefore, low levels of IL-2 favor Treg suppressive function, whereas high levels overcome suppression by favoring robust proliferation of Tconvs [33]. Importantly, a recent study using an in vivo imaging approach has demonstrated that autoantigen-driven Treg clustering with Tconvs on the surface of DCs and competition for IL-2 expressed by Tconvs is a major mechanism of autoimmune suppression [43]. A similar IL-2 competition-based suppressive mechanism has recently been reported for NK cells [44].

Tregs showed some level of proliferation in response to SA-4-1BBL stimulation. Several groups have shown that Tregs may have compromised suppressive function when actively proliferating [33]. In a clinically relevant setting where T cells were isolated from human peripheral blood lymphocytes, paraformaldehyde fixed Tregs were shown to have better suppressive function as compared with untreated Tregs in an ex vivo coculture assay [45]. Therefore, we wanted to ensure that the observed evasion of suppression is due to SA-4-1BBL directly stimulating excessive production of IL-2 by Tconvs, but not because of compromised suppressive function of Tregs arising from active proliferation. Our data show that SA-4-1BBL was able to help Tconvs evade suppression whether Tregs are proliferative or not (irradiated, but functional).

The present study demonstrating that SA-4-1BBL targets Tconvs for evasion of Treg suppression is inconsistent with a previous study demonstrating that agonists of 4-1BB receptors directly target Tregs for suppression. In particular, the authors demonstrated that an agonistic Ab against 4-1BB was effective in preventing the suppressive function of both thymic and peripheral Tregs in coculture studies by blocking the expression of IL-9 in these cells [46]. Indeed, a blocking Ab against IL-9 when used in combination with CpG-ODN induces tumor eradication, supporting the plausible role of this cytokine in immune evasion by improving Treg function. However, this study did not provide direct evidence demonstrating that Tregs are the targets of agonistic Abs to 4-1BB. The in vitro evasion of suppression data was obtained using Tconv:Treg cocultures similar to ours. Also, this study failed to demonstrate the direct role of IL-9 on Treg suppressive function in vitro. In contrast, our previous study using Tconvs and Tregs from wild type and 4-1BB KO mice provided direct evidence for Tconvs, but not Tregs, being the target of SA-4-1BBL for the observed evasion of the suppression [15].

Signaling through 4-1BB receptor plays a multifaceted role in immune regulation. The 4-1BB signaling significantly contributes to T cell activation, acquisition of effector function, and long-term memory, more so for CD8^+^ than for CD4^+^ T cells [8,9]. Similarly, 4-1BB signaling contributes to innate immune responses, involving NK cells, neutrophils, DCs, and monocytes/macrophages [9]. We and others have shown that 4-1BB signaling also impacts cells of regulatory immunity, particularly CD4^+^CD25^+^FoxP3^+^ Tregs [4,5,9,10,34,46,47]. Tregs constitutively express 4-1BB and signaling via this receptor in conjunction with TCR and IL-2R results in Treg expansion and long-term survival [6]. This positive effect on Tregs is counterbalanced by the ability of 4-1BB signaling to block the conversion of Tconvs to Tregs and the ability of this signal to overcome Treg suppression as presented in this study. In view of these findings, we hypothesize that 4-1BB signaling plays an important role in immune homeostasis following an infection. The 4-1BB-mediated upregulated expression and secretion of IL-2 and IFN-γ play a decisive role in the generation of effector immune responses and eventual homeostasis with a net outcome of clearance of infection in the absence of a collateral damage. IL-2 drives the expansion and survival of both Tconvs and Tregs, while making Tconvs resistant to suppression by Tregs. IFN-γ, on the other hand, not only prevents the conversion of Tconvs into Tregs, but also bolsters the effector function of Tconvs for clearance of infection. Once infection is cleared, the infection-induced expression of 4-1BBL on the surface of APCs declines, which in turn translates into the cessation of IL-2 and IFN-γ expression and restoration of immune suppression.

Our findings that excessive production of IL-2 by Tconvs in response to SA-4-1BBL costimulation is both necessary and sufficient in overcoming Treg suppression may have significant implications for treatment of diseases such as cancer, that are associated with an imbalance between Tconvs and Tregs. IL-2 has been used as monotherapy and in combination with chemotherapy or biologics for the treatment of cancer with significant clinical benefit for melanoma and renal cell carcinoma patients [48]. However, treatment with exogenous IL-2 is often associated with significant toxicity [49]. In this context, the ability of SA-4-1BBL to drive the expression of endogenous IL-2 in Tconvs and regulate the adaptive immune responses for the generation of effector responses is significant. The demonstrated role of SA-4-1BBL in the generation of both CD8^+^ T and NK cell effector responses against tumor [10,13], combined with its ability to prevent the conversion of Tconvs into pTregs and overcome Treg suppression, as reported herein, demonstrate the significant potential of this molecule for cancer immunotherapy and other indications where excessive frequency of Tregs contributes to pathology.

## Supporting Information

S1 File Minimal data set associated with this manuscript (Tables A-N). (PDF)
