# Supplementary material for: 4-1BB Signaling in Conventional T Cells Drives IL-2 Production That Overcomes CD4+CD25+FoxP3+ T Regulatory Cell Suppression
Source: PLoS One. 2016 Apr 6;11(4):e0153088. doi: 10.1371/journal.pone.0153088 (PMC4822835; doi:10.1371/journal.pone.0153088)
Supplement: S1 File — (PDF) [file pone.0153088.s001.pdf]

Table A. Data set corresponding to Fig 1A		
**Groups**	**Mean CPM**	**SEM**
Tconv	265.2	16.7
Treg	243	33.6
Tconv + WT APC	6880.1	1194.4
Tconv + WT APC + SA-4-1BBL	28379.6	3587.3
Tconv + 4-1BB$^{-/-}$ APC	11438.8	3117.8
Tconv + 4-1BB$^{-/-}$ APC + SA-4-1BBL	25102.3	1082.7
(Tconv + Treg) + WT APC	1538.9	109
(Tconv + Treg) + WT APC + SA-4-1BBL	12562.5	2234.1
(Tconv + Treg) + 4-1BB$^{-/-}$ APC	1309.2	280.4
(Tconv + Treg) + 4-1BB$^{-/-}$ APC + SA-4-1BBL	10456.6	819.5

Table B. Data set corresponding to Fig 1B		
**Groups**	**Mean CPM**	**SEM**
Tconv	971.9	25.5
Tconv + SA-4-1BBL	18610.3	718.4
Tconv + Treg	939.1	76.7
(Tconv + Treg) + SA-4-1BBL	32047.4	3323.6

Table C. Data set corresponding to Fig 2A		
**Groups**	**Mean CPM**	**SEM**
Tconvs + APC	15322.8	1924
Tconvs + APC + SA-4-1BBL	28230.9	5375.7
(Tconv + Treg) + APC	4126	587.6
(Tconv + Treg) + APC + SA-4-1BBL	26361.5	1413.9
(Tconv + Treg) + APC + α-TGFβ	4184	313.4
(Tconv + Treg) + APC + SA-4-1BBL + α-TGFβ	31216.1	1382

Table D. Data set corresponding to Fig 2B		
**Groups**	**Mean CPM**	**SEM**
Tconvs + APC	16056.2	2211.3
Tconvs + APC + SA-4-1BBL	34663	5403.2
(Tconv + Treg) + APC	2491.9	364.4
(Tconv + Treg) + APC + SA-4-1BBL	28560.2	948.4
(Tconv + Treg) + APC + α-TGFβ	3843.9	100.1
(Tconv + Treg) + APC + SA-4-1BBL + α-TGFβ	36892.8	3016.8

Table E. Data set corresponding to Fig 2C		
**Groups**	**Mean CPM**	**SEM**
Tconvs + APC	15322.8	1924
Tconvs + APC + SA-4-1BBL	28230.9	5375.7
(Tconv + Treg) + APC + α-IFNγ	4420.4	112.7
(Tconv + Treg) + APC + SA-4-1BBL + α-IFNγ	29271.9	407.6

Table F. Data set corresponding to Fig 3A						
	Average cytokine level in culture supernatants (pg/ml) ± SEM					
**Groups**	**IL-2**	**IL-4**	**IL-6**	**IFN-γ**	**TNF-α**	**IL-17a**
Tconv+APC	14.64±1.01	4.13±1.49	2.78±2.78	8.63±1.49	13.50±0.47	4.24±2.25
Tconv+APC+SA-4-1BBL	23.68±4.13	14.31±6.35	5.20±2.24	11.31±0.39	24.50±2.67	4.15±0.83
(Tconv+Treg)+APC	5.37	2.26	2.6±1.1	4.25±1.39	11.84	2.55
(Tconv+Treg)+APC+SA-4-1BBL	6.41±0.31	4.29±1.77	2.08±2.08	5.23±0.71	11.65±1.32	3.34±1.27

Table G. Data set corresponding to Fig 3B						
	Average cytokine level in culture supernatants (pg/ml) ± SEM					
**Groups**	**IL-2**	**IL-4**	**IL-6**	**IFN-γ**	**TNF-α**	**IL-17a**
Tconv	15.23±7.67	18.06±15.24	13.3±13.3	24.21±9.19	28.52±15.49	14.82±8.99
Tconv+SA-4-1BBL	392.8±168.3	20.17±7.97	29.6±26.5	53.85±25.65	96.40±4.41	32.75±23.46
(Tconv+Treg)	72.52±65.99	27.22±24.28	20.8±20.8	24.09±12.01	76.88±62.93	29.03±25.18
(Tconv+Treg)+SA-4-1BBL	213.22±79.82	34.83±19.58	18.7±16.6	48.53±9.23	98.70±42.01	29.47±23.64

Table H. Data set corresponding to Fig 3C		
**Groups**	**ΔΔCT**	**SD**
Tconv	1.816	1.927
Tconv + SA-4-1BBL	5.052	0.811
Tconv + Treg	1.385	1.417
Tconv + Treg + SA-4-1BBL	5.686	0.814
Treg	0.579	1.011
Treg + SA-4-1BBL	1.591	2.833

Table I. Data set corresponding to Fig 3D		
**Groups**	**ΔΔCT**	**SD**
Tconv	4.056	1.357
Tconv + SA-4-1BBL	8.808	0.608
Tconv + Treg	2.736	1.399
Tconv + Treg + SA-4-1BBL	8.892	0.373
Treg	1.743	1.600
Treg + SA-4-1BBL	4.890	0.689

Table J. Data set corresponding to Fig 4B			
Treatment		Mean CPM	SEM
hIL-2 IU/ml	0.1	376.8	14.3
	1	4368	397.2
	3	19228.2	1330
	6	55546.4	2782.7
batch 2		30870.7	2183.2
batch 3		381.4	52.7
PBS		267.9	20.3

Table K. Data set corresponding to Fig 5A		
Groups	Mean CPM	SEM
Tconv+APC	7467.5	310.7
Tconv+APC+SA-4-1BBL	19271.5	1422.2
Tconv+APC+IL-2	34859.4	2033.4
(Tconv+Treg)+APC	773.2	32.8
(Tconv+Treg)+APC+SA-4-1BBL	8540.7	703.5
(Tconv+Treg)+APC+IL-2	28613.7	333.5
(Tconv+Treg)+APC+sup w/o SA-4-1BBL	27190.3	4123.6
(Tconv+Treg)+APC+sup w/o SA-4-1BBL and IL-2	2844.2	74.5

Table L. Data set corresponding to Fig 5B		
Groups	Mean CPM	SEM
Tconv	5595.1	284.3
Tconv+SA-4-1BBL	15182	1413.7
Tconv+IL-2	30785.9	909.1
(Tconv+Treg)	6427.1	846.6
(Tconv+Treg)+SA-4-1BBL	26474.5	4410.3
(Tconv+Treg)+IL-2	52207.7	3349.3
(Tconv+Treg)+sup w/o SA-4-1BBL	31239.4	3360.7
(Tconv+Treg)+sup w/o SA-4-1BBL and IL-2	3710.85	41.6

Table M. Data set corresponding to Fig 5C	
**Groups**	**% Proliferation (CFSE)**
Tconv + APC	48.1
Tconv + APC+ SA-4-1BBL	67.5
(Tconv + Treg) + APC	18.8
(Tconv + Treg) + APC + SA-4-1BBL	64.1
(Tconv + Treg) + APC + IL-2	43.1
Treg + APC + SA-4-1BBL	11.1
(Tconv + Treg) + APC	5.3
(Tconv + Treg) + APC + SA-4-1BBL	27.3
(Tconv + Treg) + APC + IL-2	16.5

Table N. Data set corresponding to Fig 5D		
(Treg* = irradiated Tregs)		
**Groups**	**Mean CPM**	**SEM**
Tconv + APC	15889.9	2183.4
Tconv + APC + SA-4-1BBL	27760.9	1409.8
Tconv + APC + IL-2	54763.5	881.8
(Tconv + Treg) + APC	631.9	38.9
(Tconv + Treg) + APC + SA-4-1BBL	21246.6	1738.2
(Tconv + Treg) + APC + IL-2	55510.9	412
(Tconv + Treg*) + APC	5697.2	352.4
(Tconv + Treg*) + APC + SA-4-1BBL	29877.6	382.1
(Tconv + Treg*) + APC + IL-2	55754.1	2693.8
Treg + APC + SA-4-1BBL	3923.7	419.2
Treg* + APC + SA-4-1BBL	211.8	10.9
Treg + APC + IL-2	26001	630.6
Treg* + APC + IL-2	294.8	29.2
